# Elevated troponin I and its prognostic significance in acute liver failure

**DOI:** 10.1186/cc11883

**Published:** 2012-11-28

**Authors:** Vinod K Audimooolam, Mark JW McPhail, Roy Sherwood, Chris Willars, William Bernal, Julia A Wendon, Georg Auzinger

**Affiliations:** 1 Institute of Liver Studies, Department of Biochemistry, Kings College Hospital, Denmark Hill, London SE19 2RS; 2 Liver and Antiviral Centre, Imperial College London, St Mary's Hospital Campus, 10th Floor QEQM Wing, South Wharf Street, Paddington, London, W2 1NY

## Abstract

**Introduction:**

Acute liver failure (ALF) is a life-threatening multisystem illness complicated by multiple organ failure (MOF) and haemodynamic disturbances. Morbidity and mortality remains high and various prognostic and scoring models are in use to predict outcome. A recent observation in a large cohort of ALF patients suggested a prognostic value of troponin I (cTnI) and its role as a marker of subclinical myocardial injury and outcome.

**Methods:**

Data from consecutive ALF patients over a four-year period from January 2007 to March 2011 were included. The aim of this study was to correlate any relationship that may exist between cTnI, mortality, severity of illness and non-hepatic organ failure.

**Results:**

A total of 218 subjects (age 36 (16 to 90) years, M:F 103:115) were studied, of which 136 had an elevated cTnI > 0.05 μg/L. Higher organ failure scores were found with positive cTnI: APACHE II (19.5 (3 to 51) vs 14 (2 to 51), *P *= 0.001), APACHE III (81 (15 to 148) vs 59 (8 to 172), *P *= < 0.001) SOFA (15 (4 to 20) vs 13 (2 to 21), *P *= 0.027) and SAPS (48 (12 to 96) vs 34 (12 to 97), *P *= 0.001). Patients with positive cTnI had higher serum creatinine (192 μmol/l (38 to 550) vs 117 μmol/l (46 to 929), *P *< 0.001), arterial lactate (0.25, *P *< 0.001) and a lower pH (-0.21, *P *= 0.002). Also a higher proportion required renal replacement therapy (78% vs 60%, *P *= 0.006). Patients with elevated cTnI more frequently required vasopressors-norepinephrine (73% vs 50%, *P *= 0.008). Elevated cTnI did not predict outcome as effectively as other models (AUROC 0.61 (95% CI 0.52 to 0.68)).

**Conclusions:**

More than 60% of ALF patients in this study demonstrated elevated cTnI. Despite a close correlation with organ failure severity, cTnI was a poor independent predictor of outcome. cTnI may not represent true myocardial injury and may be better viewed as a marker of metabolic stress.

## Introduction

Acute liver failure (ALF) is a life-threatening multisystem illness resulting from massive liver injury. The defining clinical symptoms are coagulopathy and encephalopathy occurring within days or weeks of the primary insult in patients without pre-existing liver injury [1]. The heterogeneity of ALF in terms of underlying aetiology, its rarity and the usually progressive, severe disease course with a high fatality rate, result in limited controlled trial data being available to guide optimal therapy [2]. Although significant progress has been made over the last two decades in managing ALF patients through improvements in intensive care management, prognosis still remains poor in defined subgroups without liver transplantation. The multisystem involvement of ALF along with the unpredictable disease course poses unique challenges in managing these patients [3].

ALF is frequently complicated by progressive haemodynamic disturbances. There is evidence of peripheral vasodilatation with resultant increase in cardiac output and reduced systemic vascular resistance. Hypotension is a frequent occurrence often requiring optimal fluid loading and use of vasopressors. The pathogenesis of the haemodynamic changes are incompletely characterised but are likely multi-factorial, in part related to the systemic inflammatory response syndrome (SIRS) evoked by the liver necrosis and subsequently superimposed sepsis [4,5].

Although marked haemodynamic instability is a common finding in ALF, there is paucity of data in relation to intrinsic myocardial function in this condition. This is partly explained by the demographics of patients with ALF, in whom established risk factors for cardiovascular disease are commonly absent.

A recent study suggested that myocardial injury is frequently present in patients with ALF and demonstrated a possible association between elevated troponin I (cTnI) and outcome. The elevated cTnI was postulated to be due to subclinical cardiac injury [6]. Troponin I is a well-established, specific, and sensitive marker of myocardial injury, with both diagnostic and prognostic value. It permits early identification of patients at increased risk of death from acute coronary syndrome [7-9]. However specificity is reduced in certain conditions, such as sepsis, renal failure, pulmonary embolic disease or acute stroke, all commonly encountered in the critically ill, where cTnI levels can be elevated in the absence of an acute coronary ischemic event [9,10].

The American Acute Liver Failure Study Group focused on the relationship of cTnI elevation and outcome, but did not provide any information regarding results of diagnostic cardiac investigations such as echocardiography and/or correlation with invasive haemodynamic data.

In the present study, we analysed cTnI along with invasive haemodynamic and echocardiographic data in a large cohort of patients presenting with acute liver failure. The objectives of the study were to investigate the relationship between cTnI, hospital mortality and cardiovascular parameters derived from invasive and non-invasive testing.

## Materials and methods

All patients admitted to King's College Hospital Liver Intensive Therapy Unit (LITU) with a diagnosis of acute liver failure (hyperacute liver failure, acute liver failure and subacute liver failure) [11] between January 2007 and March 2011 were evaluated for inclusion. The data was collected from a specialist database (data collected by specialist audit staff and entered real time) within the Institute of Liver Studies, King's College Hospital (KCH). Ethical approval for analysis and publication of the fully anonymised audit dataset was given by the South East London Research Ethics Committee (formerly known as King's College Hospital Research Ethics Committee). Patients and their relatives were given the opportunity to have their dataset removed from the database; none chose to do so. Patients, who had standard blood tests including cTnI, were included in the study. A total of 278 patients with ALF were admitted during this period of which 218 (78%) patients had cTnI tested.

Admission screening tests within the first 24 to 48 h included viral serology, autoimmune screen, alpha fetoprotein, ferritin and copper in addition to full blood count, electrolytes, liver and renal function tests, coagulation screen (prothrombin time (PT) and international normalised ratio (INR)), cTnI, arterial blood gas analysis and arterial ammonia. All patients underwent 12-lead electrocardiogram regardless of troponin levels, on admission and in case of abnormalities on continuous two-lead cardiac monitoring on the Intensive Care Unit.

One hundred and forty-four patients underwent invasive haemodynamic monitoring utilising the transpulmonary thermodilution (TPTD) and arterial pulse contour techniques (PiCCO™, Pulsion, Munich Germany), based on clinical need and physician discretion. All patients who required haemodynamic monitoring had a central venous catheter placed in the internal jugular vein and an arterial catheter inserted either in the femoral artery or axillary artery. Cardiac index (CI), intrathoracic blood volume index (ITBVI (a volumetric indicator of preload) and extravascular lung water index (EVLWI) were the standard haemodynamic variables recorded. The mean of three measures of the cardiac variables on the day of cTnI testing was calculated.

Transthoracic (TTE) or a transoesophageal echocardiogram (TEE) was performed in the event of ECG abnormalities or raised cTnI. Echocardiograms were also undertaken in those patients with haemodynamic compromise with presence of shock or inappropriately low cardiac output, or if central venous pressures were significantly elevated suggesting right ventricular dysfunction. All TTE and TEE were performed by trained cardiac technicians, cardiologists or an intensive care specialist trained in perioperative/critical care echocardiography. Semi-quantitative assessment of left and right ventricular function, presence of regional wall motion abnormalities (RWMA), valvular pathology, relaxation abnormalities, elevation of right ventricular systolic pressure (RVSP) on Doppler examination and pericardial effusion were reported. All haemodynamic measures were made independently of the knowledge of cTnI results.

The presence and severity of hepatic encephalopathy was based on clinical assessment at the referring hospital or following admission to this institution. Prospective data including clinical variables for the determination of outcome was collected. Scoring models describing the severity of illness including Acute Physiology and Chronic Health Evaluation II (APACHE), Simplified Acute Physiology Score (SAPS) and Sequential Organ Failure Score (SOFA) were recorded electronically on the day cTnI was tested.

### Measurement of serum troponin I levels

Troponin I was analysed using Trop I ultra reagent supplied by Siemens Healthcare Diagnostics (Siemens AG, Munich, Germany). The ADVIA Centaur TnI-Ultra assay is a three-site sandwich immunoassay using direct chemiluminometric technology. The European Society of Cardiology and the American College of Cardiology recommend using the 99th percentile as a cutoff value for troponin assays, above which any value is considered abnormal. For the ADVIA Centaur assay, troponin levels > 0.4 μg/L are taken to represent myocardial damage, levels > 1.5 μg/L are suggestive of acute myocardial infarction. The minimum detectable concentration for this assay is 0.006 μg/L [10]. The coefficient of variation was 0.05. Patients with abnormal cTnI were divided into troponin-positive (> 0.05) or troponin-negative (< 0.05), cTnI-high (> 0.7) or cTnI-low groups (< 0.21) based on the tertiales of the distribution of cTnI results to further assess the impact of cTnI on echocardiographically derived ventricular function given that all patients with a positive troponin underwent echocardiographic examination.

### Statistical analysis

Assuming an effect size of 0.1, alpha of 0.05 and power of 80% to detect predictor independence from a model using 15 predictors, 200 sets of patient data were required. Data is reported as mean (SD) and median (range) and statistical testing utilised Student's *t *test/Mann Whitney *U *test/ANOVA/Kruskal-Wallis as appropriate following normality testing. Categorical data were compared using the χ2 test. Logistic regression and area under the receiver operating characteristic curve (AUROC) was performed to assess the effect of measured variables on outcome. Statistical significance was defined at the 95% level. Statistical analysis was performed within the Statistics Package for the Social Sciences (SPSS, version 17.0 SPSS Inc., Chicago, IL, USA) and MedCalc version 11.4.4 (MedCalc Software, Mariakerke, Belgium).

## Results

Demographic data of the study population is shown in Table 1 and 2. A total of 218 (M 103 (47%): F 115(53%)) patients who fulfilled criteria for acute liver failure were enrolled from January 2007 to March 2011. Median age was 36 years (16 to 90) and 136 (62%) patients had an elevated cTnI > 0.05 μg/L (positive cTnI) on admission. Acetaminophen intoxication was the most common aetiology for ALF with hypoxic hepatitis, viral infections and recreational drugs (cocaine (7), ecstasy (5)) accounting for the remaining cases. Non-acetaminophen drug-induced liver failure was the most common aetiology among the subacute liver failure (SALF) group followed by indeterminate and autoimmune hepatitis.

**Table 1:** The baseline variables for all patients and depending on whether a troponin I assay was positive or negative.

Variable	All patients N = 218	Troponin- positive > 0.05, N = 136	Troponin- negative < 0.049, N = 82	*P *value
Age	36 (16-90)	39 (16-80)	33.5 (16-90)	0.059

Gender (M:F)	103:115 (47%:53%)	67:69	36:46	0.529

Spontaneous survival (Y:N)	106:112	61:75	45:37	0.195

ALF/SALF	178:40	116:20	62:20	0.170

AST	2779 (22-31320)	3154 (38-20000)	2047 (22-31320)	0.0273

Bilirubin	96 (6-1265)	83 (6-739)	122 (9-1265)	0.245

Albumin	30 (16-49)	31 (18-39)	31 (16-49)	0.851

Urea	6.4 (1.5-19.2)	8.8 (2.3-19)	5.2 (1.5-15.9)	0.109

Creatinine	187 (38-929)	192 (38-550)	117 (46-929)	< 0.001

INR	4.4 (1-16)	4.3 (1-15.7)	4.4 (1.2-16)	0.649

pH	7.4 (6.9-7.5)	7.4 (6.9-7.5)	7.4 (7.23-7.5)	0.009

Lactate	2.2 (0.3-24)	2.4 (0.7-24)	2.0 (0.3-11)	0.036

CK	175 (6-37840)	292 (6-37840)	81 (14-1417)	< 0.001

CVVHF	155:63	106:30	49:33	0.006

Inotropes (Y:N)	140:77	99:36	41:41	0.008

NH4	89 (12-346)	88 (12-346)	97 (19-285)	0.545

HE grade	3 (1-4)	4 (1-4)	3 (1-4)	0.017

Heart rate	98 (45-170)	98 (45-160)	99 (50-170)	0.346

MAP	77 (45-180)	70 (50-124)	70 (45-180)	0.308

CVP	13 (4-26)	14 (5-26)	12 (4-22)	0.005

SVO2	80 (55-94)	80 (55-93)	80 (60-94)	0.365

Invasive ventilation	147:71	106:30	41:41	< 0.001

MELD	39 (11-40)	40 (11-40)	37 (12-40)	0.135

APACHE 2	18 (2-51)	19.5 (3-51)	14 (2-51)	0.001

SAPS	46 (12-97)	48 (12-96)	34 (12-97)	0.001

SOFA	14 (2-21)	15 (4-20)	13 (2-21)	0.027

Mann Whitney U test or χ2 test performed as appropriate. ALF, acute liver failure; APACHE, Acute Physiology and Chronic Health Evaluation; AST, aspartate transaminase; CK, creatinine kinase; CVP, central venous pressure; CVVHF, continuous venovenous haemofiltration; HE, hepatic encephalopathy; INR. international normalised ratio; MAP, mean arterial pressure; MELD, model for end stage liver disease; NH4, ammonia; SALF, subacute liver failure; SAPS, Simplified Acute Physiology Score; SOFA, Sequential Organ Failure Assessment; SV02, central venous saturation.

**Table 2:** Cardiovascular parameters are in general not associated with troponin positivity in this cohort other than LV dysfunction.

Variable	All N = 218	Troponin positive	Troponin negative	*P *value
Echo data available (Y:N)	144:74	100:36	44:38	0.003

	N = 144			

Abnormal ECG (Y:N)	43:101	31:69	12:32	0.712

Arrhythmia (Y:N)	7:137	7:93	0:44	0.167

LVF	16:128	15:85	1:43	0.051

RHF	8:136	8:92	0:44	0.124

Pericardial effusion (Y:N)	13:131	9:91	4:40	0.756

High PA pressures (Y:N)	8:136	7:93	1:43	0.576

RWMA (Y:N)	4:140	3:97	1:43	0.752

CI	4.3 (2-6.9)	4.3 (2-6.5)	4.2 (2.9-6.9)	0.511

ITBVI	785 (426-1366)	763 (426-1366)	735 (488-1186)	0.136

EVLWI	9.5 (4.6-34)	9 (4-34)	9 (6-18)	0.882

CI, cardiac index; EVLWI, extravascular lung water index; ITBVI, intrathoracic blood volume index; LVF, left ventricular failure; PA, pulmonary artery; RHF, right heart failure; RWMA, regional wall motion abnormality.

### Troponin-I

Patients in both the acetaminophen and non-acetaminophen-induced ALF presented with a wide range of abnormal troponin I levels ranging from 0.05 to 50, very high levels were more frequently observed in individuals with recreational drug-induced liver injury. Individuals with positive cTnI were older and had more severe organ failure. The median organ failure scores found in patients with positive cTnI were: APACHE II (19.5 (3 to 51) vs 14 (2 to 51), *P *= 0.001), SOFA (15 (4 to 20) vs 13 (2 to 21), *P *= 0.027). Patients with positive cTnI had higher median serum creatinine levels (192 (38 to 550) μmol/l vs 117 (46 to 929) μmol/l, *P *< 0.001, MWU test) and more frequently required renal replacement therapy (49% vs 22%, *P *= 0.006 χ^2 ^test). Troponin I elevation correlated significantly with pH (-0.21, *P *= 0.002) (r values), hepatic encephalopathy grade (0.25, *P *< 0.001) and arterial lactate (0.25, *P *< 0.001).

No difference in haemodynamic parameters were noted between the cTnI-negative and -positive groups for mean arterial pressure ((MAP) 70 (50 to 124) vs 70 (45 to 180); *P *= 0.221] or cardiac index (4.3 (2.0 to 6.5) vs 4.2 (2.9 to 6.9); *P *= 0.511). However patients with elevated cTnI required more vasopressor support-norepinephrine (45% vs 19%, *P *= < 0.008) Table 1. Subjects with elevated cTnI had higher creatinine kinase levels (CK) (292 (6 to 37840) IU/L vs 81 (14 to 1417) IU/L (*P *= < 0.001)) Table 1.

### CTnI and electrocardiogram

Electrocardiographic abnormalities were identified in 43 patients: 31 (cTnI-positive) and 12 (cTnI-negative) *P *= 0.712). Non-specific ST and T wave changes were diagnosed in 11 patients. Ten patients had either ST depression or elevation: Five showed evidence of ST depression, global ST changes were seen in two patients, one with pre-existing ischaemic heart disease and the other post cardiac arrest and three had ST elevation. T wave inversion was found in ten patients and eight patients had rhythm disturbances: atrial fibrillation was diagnosed in six; one patient had a re-entrant tachycardia and one showed atrial ectopics and right bundle branch block. Two patients were diagnosed with left bundle branch block. No evidence of QT prolongation was observed in this cohort.

### CTnI and echocardiography

One hundred and forty-four patients underwent echocardiography; 100 in the cTnI-positive group (TI-pos) and 44 in the cTnI-negative group (TI-neg). Thirteen patients were diagnosed with a small not haemodynamically significant pericardial effusion Table 2.

### CTnI and regional wall motion abnormality (RWMA)

RWMA were evident in 3/100 (3%) in the cTnI-positive group and 1/44 (2%) in the cTnI-negative group. There was no statistically significant association identified in regard of RWMA between cTnI-positive/negative patients (*P *= 0.752).

### cTnI and global left ventricular dysfunction (LVD)

LVD was observed in 15% of patients with a positive cTnI (15/100). Left ventricular dysfunction in the cTnI-positive group was more pronounced in those individuals with pre-existing cardiac disease such as ischaemic heart disease, cardiomyopathy and post cardiac arrest when compared to the cTnI-negative group (2%, 1/43). A borderline statistical association was noted between cTnI positivity and LVD (15/85 vs 1/43, *P *= 0.053, χ^2 ^test).

### Predictors of poor outcome in ALF/SALF

Median cTnI levels measured 0.08 μg/L (0 to 50) in those who survived and 0.18 μg/L (0 to 50) in those who either died or were transplanted; *P *= 0.046 (Mann Whitney *U *test). There was no difference in troponin positivity between the ALF (116/178) and SALF (20/40) cohort (*P *= 0.107, χ2 test), cTnI was not associated with poor outcome on either univariate or multivariate analysis (Table 3).

**Table 3:** Comparison of baseline factors for outcome.

Variable	Spontaneous survival	Died/Transplanted	*P *value
Age	34 (16-90)	38 (17-80)	0.089

Gender (M:F)	51:55	52:60	0.909

ALF/SALF	99:7	33:79	< 0.001

			

Troponin	0.08 (0-50)	0.18 (0-50)	0.046

AST	2969 (22-31320)	2197 (24-20000)	0.4122

Bilirubin	83 (6-1265)	123 (16-739)	0.011

Albumin	32 (18-49)	30 (16-38)	0.312

Urea	7.2 (2.5-19.2)	4.5 (1.5-17.9)	0.127

Creatinine	166 (38-929)	170 (46-404)	0.867

INR	3.2(1-11)	5.5 (1.5-16)	< 0.001

pH	7.4 (7.17-7.5)	7.4 (6.9-7.51)	0.074

Lactate	1.85 (0.7-24)	2.6 (0.3-19)	< 0.001

CK	151 (14-36985)	258 (6-3784)	0.210

			

CVVHF	65:41	90:22	0.003

Inotropes (Y:N)	48:57	92:20	< 0.001

			

NH4	75 (18-346)	97 (12-285)	0.001

HE Grade	2 (1-4)	4 (1-4)	< 0.001

			

Heart rate	93 (45-170)	100 (50-160)	0.051

MAP	77 (50-180)	68 (45-129)	< 0.001

CVP	12 (4-23)	14 (7-26)	0.030

SVO2	79 (60-94)	80 (55-93)	0.355

CI	4.1 (2-6.9)	4.4 (2-6.7)	0.309

ITBVI	763 (550-1186)	758 (426-1392)	0.961

			

Invasive ventilation	62:44	85:27	0.009

pO2	11.6 (7.4-26)	10.6 (5.9-19)	0.023

FiO2	0.3 (0.21-1)	0.35 (0.21-1)	< 0.001

			

MELD	35 (11-40)	40 (15-40)	< 0.001

APACHE 2	15 (2-43)	21 (3-51)	< 0.001

SAPS	36 (12-94)	51 (12-97)	< 0.001

SOFA	12.5 (2-19)	15 (4-21)	< 0.001

There was no difference in troponin positivity between the ALF (116/178) and SALF (20/40) cohort (*P *= 0.107, χ2 test), neither did troponin I predict poor outcome in either ALF or SALF cohorts so they were examined together for determinants of outcome. ALF, acute liver failure; APACHE, Acute Physiology and Chronic Health Evaluation; AST, aspartate transaminase; CK, creatinine kinase; CVP, central venous pressure; CVVHF, continuous venovenous haemofiltration; FiO2, fraction of inspired oxygen; HE, hepatic encephalopathy; INR. international normalised ratio; MAP, mean arterial pressure; MELD, model for end stage liver disease; NH4, ammonia; pO2, partial pressure of oxygen; SALF, subacute liver failure; SAPS, Simplified Acute Physiology Score; SOFA, Sequential Organ failure Assessment; SV02, central venous saturation.

Independent prognostic significance was neither found when patients were subgrouped into those with hypoxic aetiology (*P *= 0.341) nor with regards to the presence or absence of raised pulmonary artery pressures (*P *= 0.756).

Age, aetiology of liver disease, bilirubin, INR, lactate, hepatic encephalopathy, mean arterial blood pressure, continuous venovenous haemodiafiltration (CVVHF), vasopressor use and fraction of inspired oxygen (FiO2) requirements were found to be associated with poor outcome on univariate analysis. However, on multivariate analysis only bilirubin (odds ratio (OR) 1.003, confidence interval (CI) 1.000 to 1.006, *P *= 0.039), INR (OR 1.263, CI 1.096 to 1.455, *P *= 0.001), lactate (OR 1.547, CI 1.137 to 2.105, *P *= 0.005), and hepatic encephalopathy grade (OR 3.293, CI 1.895 to 5.723, *P *= < 0.001) along with organ failure scores were identified as independent predictors of poor outcome in this group (Table 4).

**Table 4:** Results of multivariate analysis of predictors of outcome.

Variable	Odds ratio	95% CI	*P *value	Odds ratio	95%CI	*P *value
Age	1.016	0.998-1.034	0.077			

Gender (M:F)	0.934	0.549-1.591	0.803			

						

Troponin	1.032	0.974-1.094	0.281			

Troponin positive	1.493	0.862-2.593	0.152			

Troponin high	1.674	0.947-2.761	0.077			

LVF (Y/N)	0761	0.271-2.174	0.617			

						

ALF/SALF	5.907	2.481-14.064	< 0.001	-	-	-

Ischaemic Aetiology	0.572	0.181-1.808	0.341			

						

AST	1.000	0.999-1.000	0.2379			

Peak AST	1.000	0.999-1.000	0.857			

Bilirubin	1.002	1.000-1.004	0.029	1.003	1.000-1.006	0.039

Albumin	0.953	0.872-1.042	0.289			

Urea	0.936	0.817-1.072	0.341			

Creatinine	0.998	0.996-1.007	0.168			

CVVHF	2.580	1.400-4.74	0.001			

INR	1.264	1.154-1.385	< 0.001	1.263	1.096-1.455	0.001

						

pH	0.037	0.001-1.407	0.064			

Lactate	1.296	1.132-1.481	< 0.001	1.547	1.137-2.105	0.005

Heart rate	1.019	1.000-1.021	0.046			

MAP	0.967	0.952-0.982	< 0.001			

CI	1.127	0.784-1.618	0.516			

ITBVI	0.996	0.997-1.001	0.708			

EVLWI	1.009	0.921-1.105	0.840			

CVP	1.108	1.009-1.214	0.025			

SVO2	1.015	0.965-1.065	0.546			

Inotropes (Y:N)	5.463	2.946-10.182	< 0.001			

CK	1.000	0.999-1.000	0.596			

						

Invasive ventilation	2.234	1.250-3.999	0.006			

pO2	0.897	0.815-0.984	0.018			

FiO2	8.392	1.502-46.79	0.009			

pO2/FiO2 ratio	0.946	0.946-0.982	< 0.001			

						

NH4	1.007	1.002-1.013	0.005			

HE Grade	2.471	1.812-3.654	< 0.001	3.293	1.895-5.723	< 0.001

						

MELD	1.130	1.071-1.198	< 0.001	-		

APACHE 2	1.081	1.042-1.124	< 0.001	-		

SAPS	1.038	1.021-1.055	< 0.001	-		

SOFA	1.168	1.088-1.254	< 0.001	-		

The multivariate analysis is for the ALF cohort only given the low numbers of SALF patients. The independent multivariate predictors are on the right. This model would correctly classify 80% of patients with an AUROC of 0.84 (0.79 to 0.89, *P *< 0.001). ALF, acute liver failure; APACHE, Acute Physiology and Chronic Health Evaluation; AST, aspartate transaminase; CI, confidence interval; CK, creatinine kinase; CVP, central venous pressure; CVVHF, continuous venovenous haemofiltration; FiO2, fraction of inspired oxygen; HE, hepatic encephalopathy; INR. international normalised ratio; MAP, mean arterial pressure; MELD, model for end stage liver disease; NH4, ammonia; pO2, partial pressure of oxygen; SALF, subacute liver failure; SAPS, Simplified Acute Physiology Score; SOFA, Sequential Organ Failure Assessment; SV02, central venous saturation.

**Figure 1:**
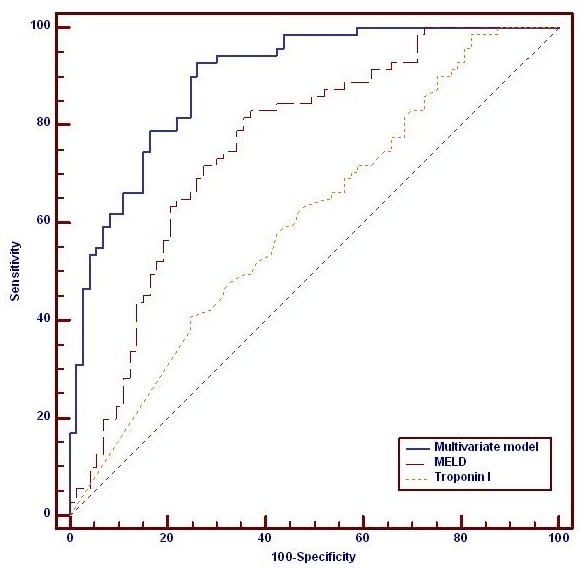
**Comparison of outcome prediction in this cohort between troponin I, MELD and a composite multivariate model using the independent variables from Table 3**. Troponin I (AUROC 0.61 (0.52 to 0.68)); MELD (0.76 (0.68 to 0.83); Composite model (AUROC 0.89 (0.83 to 0.94)), *P *< 0.001 for all comparisons. AUROC, area under the receiver operating curve; MELD, model for end-stage liver disease.

AUROC analysis demonstrated the composite multivariate model that is, bilirubin, lactate, INR and hepatic encephalopathy grade (AUROC 0.89 (0.83 to 0.94)) to be the best predictor of outcome in ALF group followed by model for end-stage liver disease (MELD) (0.76 (0.68 to 0.83)) in this mixed cohort of patients (acetaminophen and non-acetaminophen). Despite a close correlation with organ failure severity, AUROC analysis confirmed that cTnI did not predict poor outcome (AUROC 0.61 (0.52 to 0.68)) (Figure 1).

## Discussion

Elevation of cTnI in patients presenting with either ALF/SALF is not uncommon as demonstrated in the present study as well as previously by Parekh *et al *[6]. A positive correlation between cTnI elevation and vasopressor requirements, presence of acute kidney injury and organ failure scores was found; cardiac dysfunction on echocardiography or low-flow state on invasive haemodynamic monitoring was not more frequent in cTnI-positive patients. Troponin-I elevation observed in ALF/SALF is likely to represent an epiphenomenon of multi-organ failure rather than myocardial injury. Despite previous association between positive cTnI with poor outcome our data is not supportive of this.

Cardiac isoforms of TI (cTnI) and troponin T (cTnT) are highly sensitive and specific markers of myocardial injury in acute coronary syndromes [12]. However, cTnI elevations may be seen in other disease processes of cardiac and non-cardiac origin including myocarditis, pulmonary embolic disease, septic shock, renal failure and following cardiotoxic chemotherapy [13-15]. Troponin release from the myocardium into the circulation is thought to be either due to transient leakage as seen in reversible ischemia or continuous resulting from irreversible ischaemic damage [14-16]. Proposed mechanisms for troponin release in critically ill patients include focal ischemia, often triggered by massive sympathomimetic response, direct cardiac myocytotoxic effects of endotoxins, cytokines or reactive oxygen radicals due to infectious process [15-18]. In addition, it has been postulated that intracellular pathways may be triggered in critically ill patients resulting in degradation of free troponin to lower molecular weight fragments, which are released because of increased cell membrane permeability [16-18].

Several clinical and experimental studies have demonstrated a positive correlation between elevated troponin in sepsis/critical illness and unfavourable outcome. Assessment of cardiac function in sepsis and critical illness may be challenging as most of the cardiac contractility indices are affected by peripheral vasomotor tone and changes in loading conditions. In addition, catecholamine stress observed in sepsis may stimulate the myocardium and may mask myocardial depression [19]. These limitations along with the absence of a gold standard technique to assess myocardial performance have led to the utilisation of cardiac biomarkers such as troponin as sensitive prognostic markers of subclinical myocardial injury.

We found that 62% of patients with ALF/SALF had elevated cTnI. The reasons for this are likely to be multifactorial. Abnormal cTnI levels correlated closely with organ failure scores, renal failure and inotropic use. As expected, very high values of cTnI in our series were more often seen in those individuals presenting with recreational drug overdose that is, cocaine and ecstasy either in isolation or with concomitant use of acetaminophen. This may be due to direct cardiotoxic effects and or the severe metabolic derangement and high incidence of renal failure seen in these individuals.

High cardiac output associated with generalised vasodilatation is common in ALF [20]. It has been proposed that the high cardiac output state increases cardiac workload thereby increasing oxygen demand. Tachycardia can further compromise coronary perfusion due to the reduced diastolic time. In addition, the aggressive use of inotropic and vasopressor agents to maintain adequate end organ perfusion and systemic oxygen delivery may increase the incidence of cardiovascular complications. Comparison of invasive haemodynamic parameters did not demonstrate a significant difference between troponin-positive and -negative patients. This has to be interpreted with caution given the higher vasopressor and inotrope requirements in the cTnI-positive group.

Echocardiography is a readily available monitoring tool to assess myocardial performance during critical illness. A negative correlation between left ventricular ejection fraction and positive cTnI, either as a one-off measurement or performed serially has been reported in septic patients [21-24]. Myocardial depression characterised by reversible biventricular dilation, decreased systolic contractile function, and decreased response to both fluid resuscitation and inotropic therapy has been described in septic shock [25], but data in ALF is lacking.

LVD in our cohort was more often seen in patients with high troponin levels and in the context of hypoxic hepatitis due to pre-existing cardiac conditions. However, we did not observe any evidence of biventricular dilatation on echocardiography. In contrast to the sepsis literature [26], the incidence of RWMA did not correlate with cTnI levels. The lower scan frequency among the cohort with normal troponin and ECG findings is a consequence of ECHO being undertaken more often in patients with haemodynamic compromise where there was a clinical suspicion of left, right or biventricular dysfunction.

Troponin has been used has a prognostic marker in critical illness. Its role as an independent predictor of outcome has been debated in various clinical scenarios and studies. Among selected groups with sepsis, troponin elevation has been shown to be related to the severity of illness whereas in unselected critically ill patients, mortality among troponin-positive group is higher irrespective of the cause of troponin increase [23-29]. In our study, cTnI levels were lower among survivors when compared to those who either died or underwent transplantation; however neither uni- nor multivariate analysis showed a significant correlation with outcome.

## Conclusions

Troponin I elevation in critical illness is common and multifactorial. In this large cohort of patients with ALF and SALF, cTnI elevation correlated poorly with functional cardiac abnormalities observed during echocardiographic and invasive haemodynamic studies. Although higher cTnI levels were observed in patients with higher severity of illness, its value as an independent predictor of outcome was poor. Positive cTnI in this cohort is likely to represent an epiphenomenon of multiple organ failure (MOF).

## Key Messages

• cTnI elevation observed in ALF/SALF may not represent true myocardial injury and may be better viewed as a marker of metabolic stress.

• cTnI is not associated with directly measured haemodynamic abnormalities in ALF patients.

• Despite previous observations suggesting positive cTnI being associated with an independent risk of poor outcome, these data suggest cTnI positivity is an epiphenomenon of MOF that may not contribute independently to poor outcome in this cohort.

## Abbreviations

ALT: acute liver failure; APACHE: Acute Physiology and Chronic Health Evaluation; AUROC: area under the receiver operating characteristic curve; CI: confidence interval; cTnI: troponin I; CVVHF: continuous venovenous haemodiafiltration; INR: international normalised ratio; LVD: left ventricular dysfunction; MOF: multiple organ failure; MELD: model for end-stage liver disease; OR: odds ratio; PT: prothrombin time; RWMA: regional wall motion abnormality; SD: standard deviation; SALF: subacute liver failure; SOFA: Sequential Organ Failure Assessment.

## Competing interests

The authors declare that they have no competing interests.

## Authors' contributions

VKA carried out analysis interpretation and drafted the manuscript. MJWM carried out the statistical analysis. GA, JAW, CW and WB carried out the correction of manuscript for important intellectual content. RS participated in the biochemical analysis. All authors have read and approved the manuscript for publication
